# Molecular simulations of self-assembling bio-inspired supramolecular systems and their connection to experiments

**DOI:** 10.1039/c8cs00040a

**Published:** 2018-04-24

**Authors:** Pim W. J. M. Frederix, Ilias Patmanidis, Siewert J. Marrink

**Affiliations:** a Groningen Biomolecular Sciences and Biotechnology Institute and Zernike Institute for Advanced Materials , University of Groningen , Groningen , The Netherlands . Email: p.w.j.m.frederix@rug.nl ; Email: s.j.marrink@rug.nl

## Abstract

In bionanotechnology, the field of creating functional materials consisting of bio-inspired molecules, the function and shape of a nanostructure only appear through the assembly of many small molecules together. The large number of building blocks required to define a nanostructure combined with the many degrees of freedom in packing small molecules has long precluded molecular simulations, but recent advances in computational hardware as well as software have made classical simulations available to this strongly expanding field. Here, we review the state of the art in simulations of self-assembling bio-inspired supramolecular systems. We will first discuss progress in force fields, simulation protocols and enhanced sampling techniques using recent examples. Secondly, we will focus on efforts to enable the comparison of experimentally accessible observables and computational results. Experimental quantities that can be measured by microscopy, spectroscopy and scattering can be linked to simulation output either directly or indirectly, *via* quantum mechanical or semi-empirical techniques. Overall, we aim to provide an overview of the various computational approaches to understand not only the molecular architecture of nanostructures, but also the mechanism of their formation.

## Abstract 2

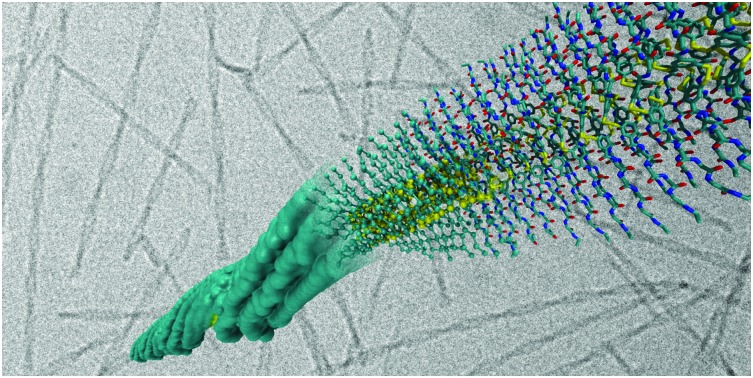
The self-assembly of bio-inspired supramolecular polymers can be unravelled using molecular dynamics simulations combined with experiments.

## Introduction

Self-assembly, also known as supramolecular polymerisation, is an extremely common phenomenon in Nature; the formation of membranes, complexation of proteins or even cell–cell interactions all occur through non-covalent interactions. The notion that biomolecular systems can be used to enhance materials design for *e.g.* medical technology has therefore taken a firm place in chemistry.^1^ Small bio-inspired molecules, such as lipids or short peptides have received particular attention: they belong to a class of molecules that are easy to synthesize or modify, have a wide chemical diversity and an inherent biocompatibility. Since the first examples and applications in the 1990s,^2^,^3^ countless variations of peptides, lipids and combinations thereof, termed peptide amphiphiles (PAs), have been shown to organise themselves into nanostructures with various properties and functions. Commercial applications have been developed in cell culture, medical technology and wound healing, among other fields. Another class of biocompatible materials is that of supramolecular light-harvesting structures, such as dye aggregates that mimic natural photosynthetic systems or can be used in organic photovoltaics.

Much of the progress in the field hitherto has been based on trial and error, though slowly but surely researchers are starting to discover the general design principles that govern molecular self-assembly. Finding quantitative (molecular) structure–property relationships (QSPR) in the context of meso–macroscale properties is an exciting endeavour that is still much more in its infancy than QSPR for protein–drug binding, for example. The theory of self-assembly from a thermodynamic point of view is useful to predict self-assembly from knowledge of the partition function landscape underlying the aggregation. This can be successfully applied to well-defined assemblies such as DNA origamis or metal–organic frameworks, as reviewed by Palma, Cecchini and Samorì.^4^ The main difficulty, however, still lies in the conformational flexibility of small molecules that causes a multidimensional and shallow intermolecular potential energy surface where local disorder is often prominent and entropy and enthalpy continuously interchange.

Experimental techniques lack the spatial resolution to inform on the exact conformation of the individual building blocks. Typically, this means the packing of molecules can only be explained by a combination of many experimental techniques. The sparse nature of the nanostructures in their solvent often precludes common structural determination techniques such as X-ray crystallography or solution-phase NMR. Less information-dense experiments that inform on small parts of the molecular conformations are combined to collect a complete picture of intermolecular interactions. This often involves measurement of light absorption (UV/Vis, infrared (IR) and circular dichroism (CD)) and scattering (dynamic and static light scattering (DLS/SLS), wide or small angle X-ray scattering (WAXS/SAXS), small angle neutron scattering (SANS)). Analysis of these experiments is performed and put besides atomic force or electron micrographs to arrive at a model of molecular interactions.

This approach is limited in three ways: firstly all techniques mentioned above are measuring ensemble averages of molecular conformations. The potentially important disordered component is therefore often neglected and leads to false models of idealized interactions for every molecule. Secondly, interpretation of the raw data occurs *via* empirical models developed for macromolecular systems. For example, IR absorption and CD of peptides are often compared to models for proteins, which are not generally valid. Finally, the timescale of self-assembly spans nanoseconds to weeks. While macroscale lab experiments can often deal with the upper half of this range, the initial aggregation of molecules is difficult to probe due to both sensitivity and time resolution issues. This precludes obtaining information on the early stages of the mechanism of molecular assembly. This important limitation was also appreciated by the community researching purely biologically relevant peptide or protein aggregation (*e.g.* amyloidogenic sequences) and it is now understood that the early stages of assembly, also known as the nucleation stage, are crucial for the final outcome and toxicity of biological aggregates.^5^

**Fig. 1:**
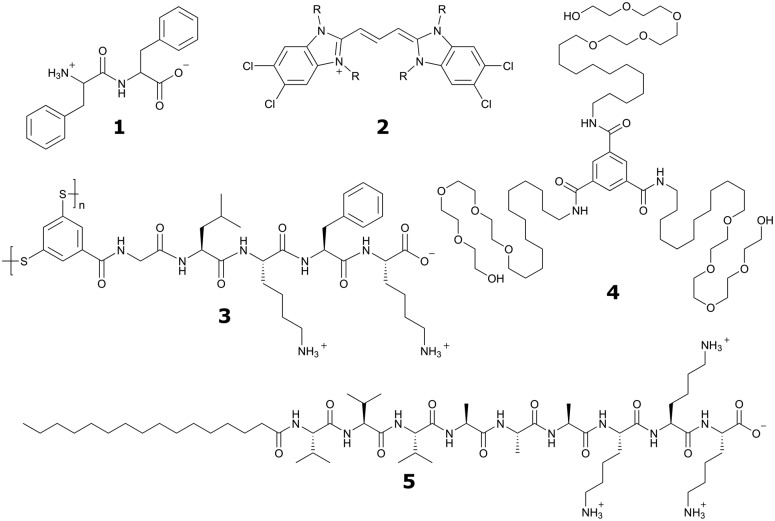
Chemical structures of popular classes of bio-inspired self-assembling molecules that have been studied using both experimental and computational techniques and will be discussed in this review. Short peptides, including phenylalanine dipeptide, **1**. Amphipathic dyes using the 5,5′,6,6′-tetrachlorobenzimidacarbocyanine core motif, **2**. Self-replicating macrocyclic peptides, including X-GLKFK, **3**. Benzenetricarboxamide (BTA) derivatives, **4**. Peptide amphiphiles, including palmitoyl-Val_3_Ala_3_Glu_3_, **5**.

One way to overcome these barriers is to use classical molecular simulations. This computational method tracks the motion of individual atoms or molecules over time, thereby negating ensemble averaging and mechanistic limitations. For the last decade simulations have added valuable insights to various self-assembly processes as has recently been reviewed.^6^–^12^ However, molecular simulations have often been used as ‘another piece of the puzzle’, working separate from the experimental techniques to qualitatively add to the knowledge about a particular supramolecular polymer. In this review, we attempt to discuss the next step, *i.e.* using the knowledge built up over this decade to (1) design new self-assembling biomolecular systems using *in silico* methods and (2) directly validate spectroscopic or scattering results by linking molecular simulation output directly with experimental observables. To achieve this we discuss recent progress in molecular simulations, including enhanced sampling techniques, before proceeding to review specific research that explicitly links the computational and experimental work, both qualitatively or quantitatively *via* semi-empirical or quantum mechanical calculations. We focus on short peptides, amphipathic dyes, designer PAs and other bioconjugates developed for materials purposes, as summarized in Fig. 1. These molecules give rise to a large variety of morphologies, including nanofibres, tubes and vesicles that function as hydrogels, light-harvesting complexes or drug delivery vehicles, among others. For reviews of computational efforts in combination with experiments on purely biological fragments such as amyloidogenic peptides, lipids, or DNA origami, please consider ref. 13–17.

## Molecular simulation techniques

**Fig. 2:**
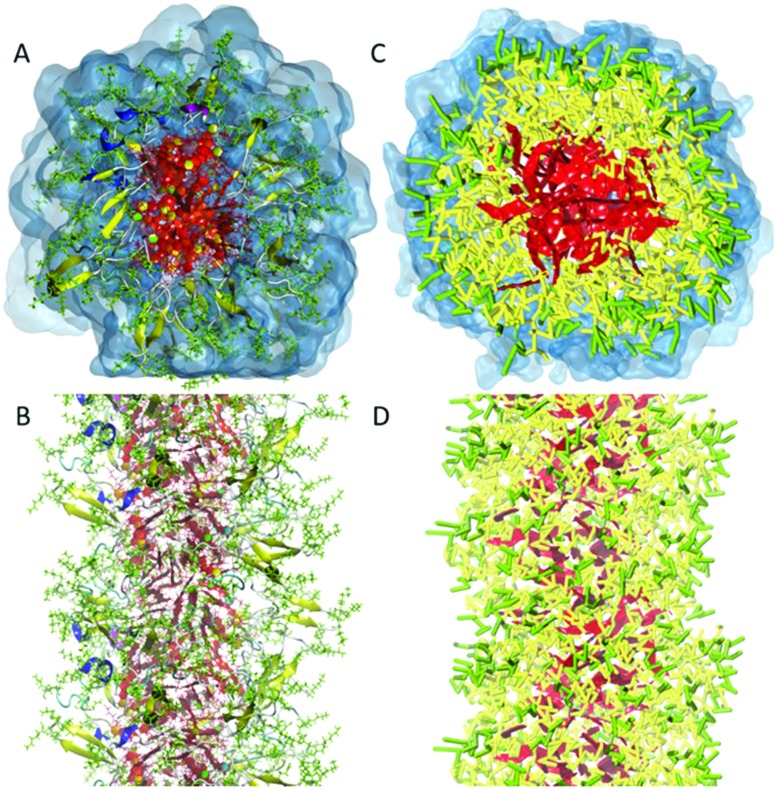
All-atom *versus* coarse-grain model of a drug-amphiphile filament. The drug-amphiphile consists of a hydrophobic cancer drug camptothecin (CPT) connected to a β-sheet-forming peptide sequence (CGVQIVYKK) *via* a short linker. (A and B) Top and side views of an all-atom representation of the filament, with CPT shown in red, and peptide residues in green. The β-sheets formed along the length of the filament are shown in yellow. (C and D) CG representation, with CPT also depicted in red, the charged lysine and polar glutamine groups shown in light green, while the other residues shown in yellow. Hydrating water is shown in the top views (A and C) only. Reproduced from ref. 19 with permission from The Royal Society of Chemistry.

In the process of supramolecular self-assembly individual molecules are coming together *via* non-covalent interactions. The most widely used simulation technique to study this process is molecular dynamics (MD). In MD, Newton's equations of motion are solved for a system of interacting particles. The interactions are described using classical force field terms which typically contain bonded potentials to model the intra-molecular interactions, and non-bonded potentials (*e.g.*, Lennard-Jones, Coulomb) to model inter-molecular interactions. Solvent may either be represented explicitly or implicitly. In the latter case, stochastic integration techniques, such as Brownian dynamics (BD) and dissipative particle dynamics (DPD) are used. In the case of discontinuous MD (DMD), the equations of motion are solved on a grid for computational speedup. Before running a simulation, a starting structure is generated, either based on a randomized placement of the constituents (*e.g.*, to simulate self-assembly) or by pre-arranging them into the desired morphology (*e.g.*, fibre, vesicle). From the starting configuration, usually some energy minimization and equilibration runs are performed to relax the initial state. Simulated annealing, in which the temperature of the system is temporary increased in a number of cycles, is a popular way to speed up the equilibration phase. Traditionally, MD and related techniques treat all atoms (AA) as interaction centres. To observe formation and equilibration of supramolecular structures, however, all-atom models often prove inadequate. To this end, coarse-grain (CG) force fields, that unite groups of atoms into effective interaction centres, provide a powerful alternative (Fig. 2). A variety of enhanced sampling methods exist to further increase the scope and accuracy of the simulations. Below we give the state-of-the-art of current AA and CG simulations as well as enhanced sampling techniques applied in the field of supramolecular aggregates. Note that when quantum mechanical (QM) effects or covalent bond formation/breakage need to be treated with higher accuracy, *ab initio* MD protocols could be employed. However, their computational cost has thus far limited them to intramolecular interactions, small molecular aggregates and crystals. For a discussion of the progress using these methods, please consider the review of Li and co-workers.^18^

### All-atom force fields

Force fields with full atomistic detail are the most suitable choice to study the exact conformation of individual building blocks inside a supramolecular aggregate, as they are generally able to accurately capture H-bonding as well as aromatic and charge–charge interactions by representing them *via* the Coulomb and Lennard-Jones potentials. The force field parameters can be obtained from quantum-mechanical (QM) calculations, together with a variety of experimental data. The ongoing development of AA force fields such as AMBER,^20^ CHARMM,^21^,^22^ OPLS^23^ and united atom force fields (implicit aliphatic hydrogens) such as GROMOS^24^,^25^ has strongly contributed to their popularity in bio-inspired molecular simulations. Solvent-free versions are available for most of these force fields, allowing a significant increase in computational efficiency, in particular for self-assembling systems, at the cost of a reduced accuracy. There seems to be no clear preference of the community which of the atomistic force fields are most suitable for simulating the self-assembly of bio-inspired molecules. Comparative studies are sparse in this context. An exhaustive set of MD simulations on the folding of tetra- and pentapeptides, performed using CHARMM, AMBER, as well as OPLS, showed clear inconsistencies between these force fields.^26^ A similar observation was made in a study on the dimerization propensity of amino acid side chain residues.^27^

#### Packing motifs

Keeping the above considerations in mind, AA simulations have contributed a lot toward our understanding of the packing motifs of supramolecular assemblies. Typical system setups range from small-scale studies on monomer–monomer interactions, to larger scale self-assembly simulations and the study of pre-formed aggregates. At the smallest scale, *i.e.* monomers and dimers, AA simulations are mostly used to improve or validate the underlying force field. For instance, Bejagam *et al.* developed an AA model for 1,3,5-benzenetricarboxamide (BTA) using QM calculations of gas phase dimers as a reference.^28^ The energy and structure of the BTA dimer at the QM level could be captured by the AA model. The AA model was then used to study dimerization in nonane, and a dimer free energy of 13 kcal mol^–1^ was obtained. Attachment to a pre-constructed fibre was found to be more favourable, demonstrating cooperativity of the process. Another nice example of force field development and validation is the work of Sasseli *et al.*^29^ The authors describe a systematic parameterization of the Fmoc moiety using CHARMM, based on QM data (monomer in solvent, dimer, torsional potentials) as well as experimental octanol/water partitioning coefficients. Validation of the model came from self-assembly of different types of Fmoc-peptides in water, one forming fibres, the other spherical aggregates, both in agreement with experiment. In addition to force field development, small scale simulations can be very useful to detect possible binding and stacking modes of the building blocks.^30^ However, the question always remains whether these modes are representative for the self-assembled state.

#### Initial self-assembly pathways

The essence of supramolecular systems is that they form *via* self-assembly. To capture this process in AA simulations is not trivial, given the limits in time and length scales that can be achieved. The time scales explored are usually limited to the nanosecond range, with system sizes typically containing a few 100 monomers. Most of the AA self-assembly simulations reported to date are therefore not leading to well-defined structures, but often result in the formation of small irregular aggregates. Despite this caveat, self-assembly simulations are important for showing possible stacking motifs inside the aggregate and revealing the driving forces of the initial growth process. Quite a number of studies deal with the self-assembly of short peptides^31^–^35^ or peptide conjugates.^36^–^38^ Already such simple systems give rise to a rich behaviour in terms of the initial self-assembly kinetics, exemplified by the formation of unexpected alpha-helical intermediate structures of beta-fibril forming peptides,^31^ and the strong effect of peptide rigidity on the order inside the aggregates formed.^33^ To further complicate the issue, the driving forces appear highly system-dependent. The π–π-stacking was found to be important in driving the initial self-assembly of peptide–drug conjugates into fibres,^36^ whereas fibre formation in the absence of aromatic residues was observed for other small peptides.^32^ One of the most interesting AA studies on self-assembly has been reported by Garzoni *et al.*^39^ They studied the mechanism of growth of BTA fibrils in aqueous conditions. Based on simulations of self-assembled short non-equilibrium aggregates of different length, estimates of the monomer ‘free energy’ in stacks of different length could be obtained. The results indicate a cooperative mechanism for supramolecular polymer growth, where a critical size must be reached in the aggregates before emergence and amplification of order into the experimentally observed fibers. Detailed analysis of the simulation data suggests that H-bonding is a major source of this stabilization energy. The work provides evidence for the key driving force of hydrogen bonding in enhancing the persistency of monomer stacking and amplifying the level of order into the growing supramolecular polymer. Studies into molecular driving forces can even have practical impact in popular culture such as avant-garde cuisine, as the ‘spherification’ of liquid food on the nanoscale was found to be driven by calcium-induced aggregation of alginate.^40^

**Fig. 3:**
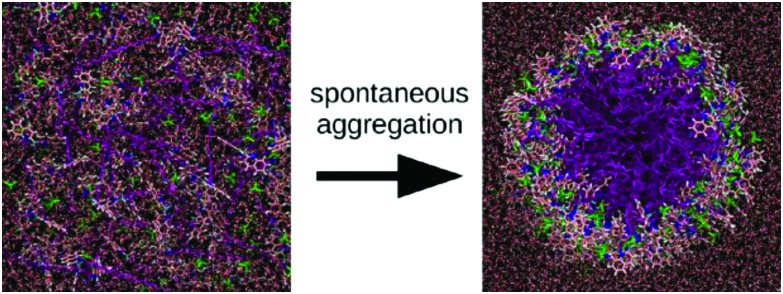
Self-assembly of amphiphilic cyanine dyes into tube structure. The dye molecule is amphi-pseudoisocyanine, a cyanine dye with two hydrophobic tails (CH_3_ and C_18_H_37_). The dye molecules are depicted with the tails purple; the nitrogen atoms are dark blue; the aromatic carbons are pink; the linker between the aromatic rings is light blue; and hydrogens connected to aromatic carbons are white. Perchlorate counterions are shown in green. For the water molecules, oxygen atoms are red and hydrogen atoms are white. Figure adapted with permission from ref. 41. Copyright 2013 American Chemical Society.

A nice example of the difficulty that simulations at the all-atom level face to obtain equilibrated self-assembled structures is provided by the work of Haverkort *et al.*^41^ They performed extensive self-assembly simulations of amphi-pseudoisocyanine, a dye that forms highly ordered, single-walled cylindrical J-aggregates in experiment, see Fig. 3. The simulated structure, although in the correct morphology, contained a high amount of disorder, despite multiple cycles of simulated annealing. Note, however, that the precise level of disorder is not known, as this is difficult to assess experimentally. It is conceivable that the idealized structures often used to illustrate the packing of molecules inside fibres are unrealistic and overly ordered.

#### Idealized starting structures

The lack of knowledge about the packing details of the supramolecular aggregates is one of the main reasons to resort to all-atom simulations. The idea of this so-called “top-down” simulation approach is that, starting from idealized structures, simulations provide a more relaxed and dynamic picture, and may discriminate between alternative structural models. Experimental constraints (*e.g.*, overall morphology, or diameter and pitch length in case of fibrils) are used to create the starting configurations. This, by itself, is already far from trivial. Packing molecules inside a predetermined shape (*e.g.*, bilayer, fibril, tube) at the correct density, without creating unphysical overlap between molecules, is not straightforward. Smart ways of generating the starting structures such as the new rolling procedure proposed by Sevink *et al.* might help,^42^ but are not generally applicable. In practice, this means that the initial configurations are under a lot of stress, leading either to numerical instabilities or causing distortion or even disintegration of the aggregate. Careful minimization and equilibration runs are therefore usually required, The work of Megow *et al.*^43^ on J-aggregates provides a nice example of this procedure. They use different wrapping and packing motifs to create aggregates of tetrachloro-tetraethyl-benzimidacarbocyanine (TTBC) dyes. The most stable fragments are then selected in an iterative process to obtain a final stable aggregate matching the experimental morphology, a single-walled tube. The same authors also report a relaxed structure of a double-walled tube composed of C8S3 dye molecules.^44^ Another example is the work of Ortony *et al.* on PA fibres (palmitoyl-Val_2_Ala_2_Glu_2_).^45^ Here the initial model was built using the software tool Packmol,^46^ a program that can pack molecules into arbitrary shapes. The resulting structure was subsequently annealed during 200 ns, followed by a 600 ns equilibration run. The critical choice of the initial structure is further illustrated in the work of Iscen and Schatz,^47^ who reported that the amount of beta strands observed in PA fibres is not only dependent on the temperature, but also on the initial packing density. To make the situation even more challenging, often different packing motifs are consistent with the experimental data. To discriminate between such models, the most used criterion is overall stability of the aggregate. This is usually measured by monitoring the potential energy of the aggregate, or by analysing the overall order of the system. Examples in the literature are plenty, *e.g.* pertaining BTA tubes,^48^ PA fibres,^49^–^51^ and short peptides.^29^,^52^,^53^ In case more than one model proves stable in the simulations, further analysis and comparison to experiments is needed to discriminate between them (see section Comparison to Experiment). Still, sometimes one ends up with different models that all fit to the experimental data equally well, as in the study on fibres composed of peptide macrocycles.^54^ Assuming a well-converged and realistic structure, AA simulations can be further used to explore the effect of state conditions (temperature, pH, ionic strength) on the properties of the aggregates,^55^,^56^ or to explore the effect of substituents or additives.^38^,^57^,^58^

#### Challenges for all-atom approaches

To bring the field further ahead, the current challenges considering all-atom force fields are threefold. First, there is room for further improvement of the AA force fields. Interactions with water and partitioning between hydrophilic and hydrophobic environments should be key targets in their parametrization as these always play a relatively large role in the process of self-assembly. Problematic, however, is the unavailability of solid experimental data on partitioning free energies of some of the key building blocks of supramolecular materials, as in the case of porphyrin based compounds.^59^

Another important target for force field optimization is reproducing crystal packing and melting data. The stacking interactions of aromatic residues in supramolecular polymers, after all, are not too dissimilar from those found in the crystal environment. Adams *et al.* explicitly considered crystal *versus* gel packing energetics in the design of efficient gelators.^60^ In some cases, in particular considering electrostatic interactions between conjugated systems, polarisable models^61^–^66^ may be required. Including some electronic degrees of freedom, polarisable models are potentially more accurate, but also more expensive computationally. Applications in the area of supramolecular self-assembly are therefore still lacking. Another important development is that of constant pH models. The p*K*_a_ of self-assembling molecules is often such that either a fully protonated or a fully deprotonated state is unrealistic. Moreover, the p*K*_a_ may shift depending on the local environment of the monomer.^67^ Efficient constant pH algorithms are therefore likely to be of considerable interest.^68^,^69^ A second challenge is to improve on tools to streamline the MD workflow. Automated topology builders (CgenFF,^70^,^71^ SwissParam,^72^ LigParGen,^73^ PRODRG,^74^ ATB,^75^ Charmm-GUI,^76^ H++,^77^ among others) are playing an increasingly important role in the opportunity to rapidly set-up self-assembly simulations and screen larger groups of input molecules. Extensions to deal with more complex, multi-component systems for a wide range of morphologies are still needed. The third challenge is to extend sampling. At the all-atom level, self-assembly and growth of aggregates is difficult to observe – either formation of a nucleus is required, or one gets easily trapped in disordered aggregates that take a long time to heal. Relaxation of pre-assembled structures is also difficult. The starting structures might thus put a bias to the system, and often this is not known. Continuous improvement of computer hardware, and the ability of most common MD software packages to run efficiently on large numbers of compute nodes including GPUs, alleviates some of this problem, but it is unlikely that AA simulations are capable of dealing with the hierarchy of length and time scale underlying supramolecular self-assembly in the decades to come.

The above caveats also limit the predictive power of AA simulations when searching for new self-assembling systems. For peptide monomers, some MD approaches have resulted in the classification of peptide building blocks towards foldability^26^ and predicted secondary structure.^78^,^79^ However, prediction of the packing of many molecules together in larger nanostructures (dimensions greater than a single foldamer) is not straightforward from recognition motifs. To some extent, AA MD assembly simulations can be used to screen among self-assembly candidates in a high-throughput fashion as demonstrated by Smadbeck *et al.*^34^ They developed a hierarchical framework for multimeric assemblies based on computing the interaction potential energy, fold specificity and approximate association affinity of 128 tripeptide sequences, while taking into account experimental constraints and design templates. They discovered multiple new self-assembling systems as verified by experiments. Importantly, Gupta, Adams and Berry were able to predict the gelation behaviour of aromatic peptide conjugates.^80^ They employed a large experimental training set of both gelling and non-gelling dipeptides with various aromatic capping groups to derive QSPR. They found the most important physicochemical descriptors were the number of rings, predicted molecular aqueous solubility, polar surface area, solvent accessible surface area, *A* log *P* and number of rotatable bonds and were even able to obtain 100% accuracy in their validation set of 9 new molecules in terms of gelation behaviour. MD simulations of assembly could definitely expand the scope of such a method without laborious experiments for every new class of building blocks, but are currently not feasible at the AA level. Two potential solutions are to resort to coarse-grain force fields and to utilize enhanced sampling methods, as will be discussed below.

### Coarse-grain force fields

As mentioned, self-assembly can take place on various time and length scales. Routine AA MD simulations are currently limited to approximately 10^6^ particles for systems that need to be simulated for 100s of nanoseconds. A cubic system containing pure water would thus be restricted to 22 × 22 × 22 nm^3^. Some nanostructures may be smaller than this, but in most cases at least one of their dimensions exceeds this limit, especially considering experimental low mM concentrations of monomers. Moreover, structural order often does not converge on nanosecond timescales and the need for longer simulations is omnipresent. Coarse-graining the degrees of freedom of the constituents of the simulation is a popular solution that can extend the use of simulations well beyond these limits.^81^ However, in particular in the field of supramolecular polymers, it is important to retain the chemical specificity of the building blocks, as it has often been shown that small modifications, such as amino acid mutations, can have a dramatic effect on their self-assembly. Several coarse-grain (CG) force fields have been developed along these lines to study self-assembling systems with chemical specificity.

A CG force field that has been used by several research groups for a variety of bio-inspired systems is the Martini force field.^82^–^85^ Originally developed to study lipid membranes, the force field has been extended to encompass all major classes of biomolecules as well as other classes of soft matter (polymers) and nanoparticles (*e.g.*, fullerene, carbon nanotubes, gold particles). The non-bonded interactions in the force field are parametrized targeting partitioning free energies of building blocks between water and non-polar solvents, which is also a key concept in the assembly of amphiphiles. This parametrization strategy combined with the inherent versatility and compatibility of the various classes of molecules developed makes the Martini force field particularly attractive to study supramolecular systems. The Martini model relies on a four to one mapping scheme, *i.e.*, four heavy atoms together with associated hydrogen atoms are represented by a CG site (also denoted CG bead). For rings, a higher resolution is used. The interactions between the CG sites are calibrated with respect to experimental thermodynamic data as well as reference AA simulations. Compared to all-atom force fields, the Martini model typically obtains a speed up of three orders of magnitude. In addition, the dynamics of most processes are faster due to an overall reduced friction. This makes the interpretation of time scales somewhat problematic, and typically only provides an order of magnitude estimate of the real dynamics. Another important limitation is the inability of the Martini model to account for secondary structure transitions in longer peptides and proteins, due to the lack of directional hydrogen bonding terms in the force field. Usually the secondary structure is fixed using an elastic network^86^,^87^ that can be further optimized with respect to atomistic simulations.^88^ Other CG force fields that are applied in the field of supramolecular self-assembly are the Shinoda–Devane–Klein (SDK) model,^89^ which is rather similar in mapping and parameterization strategy to Martini, and the PRIME model,^90^ a CG protein force field allowing secondary structure changes and making efficient use of discontinuous MD (DMD). In principle, more accurate CG models can be derived based on force matching, iterative Boltzmann (IB) and related procedures.^91^–^95^ These approaches aim to capture the higher order correlations of AA reference simulations in effective CG pair potentials. The advantage over generic force fields such as Martini is their higher accuracy, in particular with respect to structural detail. A disadvantage of these models is that they are usually less transferable and require more parameterization effort, plus the need for smaller time steps to integrate the equations of motion, thereby reducing the computational efficiency.

#### Large-scale self-assembly

Perhaps not surprisingly given the limitation of AA models, the main application of CG models with respect to bio-inspired supramolecular aggregates has been to study the self-assembly and growth process. The dynamics of thousands of monomers can be followed over time scales of tens to hundreds of microseconds. Very popular building blocks that give rise to a large variety of supramolecular structures are short peptides (2–10 residues) and peptide conjugates. Based on CG simulations, many groups study their self-assembly process aiming to identify possible intermediates and to explore the effect of state conditions on the preferred morphologies. Most of these studies use the Martini force field^96^–^103^ following the pioneering work of Frederix *et al.*^104^ Other CG force fields such as SDK^19^ and PRIME^105^ as well as dedicated CG potentials obtained with IB^106^–^108^ are also used. A few examples that use CG simulations combined with experiments will be discussed in the section “Comparison of Simulation and Experimental Results”.

**Fig. 4:**
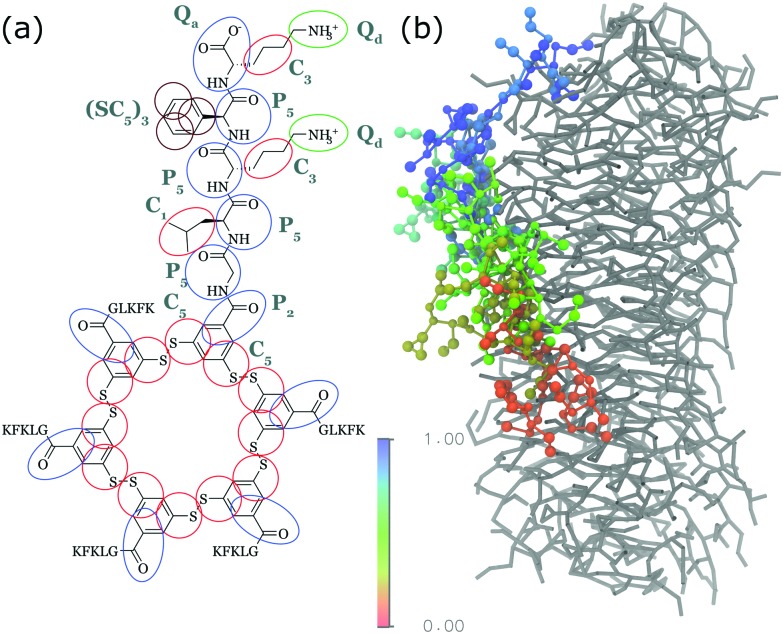
(a) CG Martini mapping of a DCL peptide macrocycle as described in ref. 112. Atoms are grouped into beads as indicated by the circles. Different colours indicate different bead types as labelled. Only one of the 6 arms of the macrocycles is shown explicitly. (b) CG simulation of the growth of a DCL fibre (in grey). A new monomer is added to the solution around the fiber, lands on the side of the fiber at time = 0 μs (red) and diffuses over 1 μs towards the end of the fiber, promoting growth.

Apart from peptide-based compounds, only few CG studies on other systems have been reported to date. A possible reason is the difficulty to obtain reliable CG models for more complex molecules often involving conjugated ring systems. Exceptions are the CG self-assembly simulations on synthetic constructs from trimaleimide functionalized with peptide linkers and cholesterol, forming nanosponges,^109^ and of porphyrin/dipeptide mixtures using DPD.^110^ A recent highlight is the work of Bochicchio and Pavan who parameterized a Martini model for BTA.^111^ To improve the description of the stacking interactions between the BTA cores, additional charged particles were introduced to mimic hydrogen bonding. The model was further optimized and validated by comparison to all-atom data. Subsequent simulations provide a picture of a stepwise cooperative polymerization mechanism, where initial fast hydrophobic aggregation of the BTA monomers in water is followed by the slower reorganization of these disordered aggregates into ordered directional oligomers. Supramolecular polymer growth then proceeds on a slower time scale. The use of a CG model allowed also for a systematic investigation of the effect of structural variations and temperature on this process. Another example of supramolecular polymer growth is provided in our own lab, based on cyclic macrocycles.^112^ Using an optimized Martini model, we observe the growth of a macrocycle fibre when individual building blocks are added in solution (Fig. 4). The growth proceeds by adsorption of molecules at the body of the fibre, followed by 1D diffusion along the fibre to reach the tip where the new building blocks attach. This growth process is supported by high-speed AFM experiments.

#### High-throughput simulations

In addition to the ability to simulate self-assembly and growth, CG models are also ideally suited for high-throughput applications. A showcase example is the work of Frederix *et al.* who simulated all 8000 combinations of tripeptides using the Martini force field in an effort to understand the design rules that govern short peptide assembly.^113^ From the *in silico* screening they were able to successfully predict self-assembling and non-assembling sequences as verified by a full experimental characterization of the top candidates resulting from the simulation protocol. Importantly, this approach also resulted in guidelines for new peptide materials by noting the asymmetric contributions to aggregation propensity of certain amino acids in particular positions of the peptide (near N- or C-termini). In an extension of this work, the co-assembly of di-, tetra- and hexapeptides was investigated to help the interpretation of experiments using dynamic combinatorial libraries where mixtures of peptides are present.^114^ The simulations suggest that co-assembly can be disruptive to the formation of larger aggregates, and that the addition of one peptide can affect the aggregation behaviour of another one. In addition to building block structure, it is also possible to use high-throughput simulations to screen the experimental conditions that are most suitable for a specific molecule to assemble. An example is the DMD study of Fu *et al.* based on the PRIME force field.^115^ Considering many independent simulations involving 800 PAs (palmitoyl-Val_3_Ala_3_Glu_3_), the authors could construct a phase diagram showing distinct nanostructures formed as a function of temperature and hydrophobic strength. One can imagine this approach can be extended to other factors commonly affecting self-assembly, such as pH, ion nature and concentration.

#### Challenges for coarse-graining

**Fig. 5:**
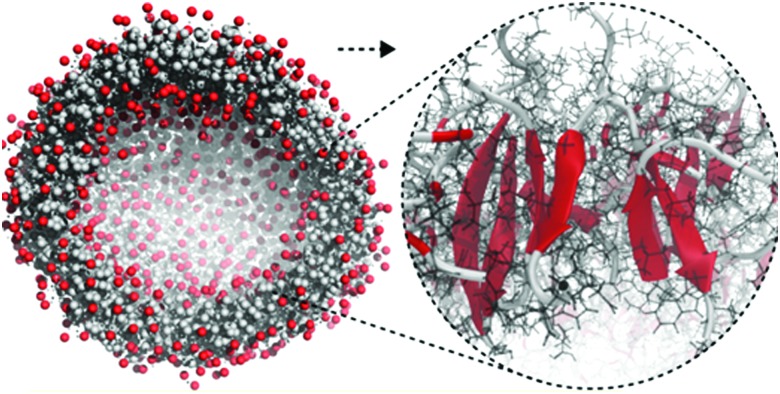
All-atom to coarse-grain backmapping example. CG structure of a self-assembled amphiphilic peptide nanovesicle (left), and zoomed view of the all-atom structure obtained after backmapping (right). In the left image, hydrophilic/hydrophobic residues are coloured red/grey. In the right image, red colour is used to indicate extended secondary structure formation. Figure adapted with permission from Rad-Malekshahi *et al.*^126^ Copyright 2015 American Chemical Society.

To further increase the potential of CG models, there are a few challenges. The first is to generate better tools for automatic topology building and system set-up, to facilitate high-throughput applications. The first steps in this direction are already taken with tools such as the Martini ATB,^116^ and CHARMM-GUI Martini maker,^117^ and the VOTCA package^118^,^119^ to construct IB and related CG potentials, but more versatility and validation is needed for these tools to be applicable to the type of supramolecular systems considered here. Another challenge is the combination of CG and AA models, *i.e.*, multiscaling. Several tools are available to switch in resolution between AA and CG and *vice versa*, so-called serial multiscaling, resolution transformation, or backmapping.^120^–^123^ This is a powerful approach to validate self-assembled structures obtained at the CG level with (short) AA simulations (Fig. 5), or to obtain higher resolution structures for details of local packing, analysis of H-bonds or subsequent use in QM calculations.^124^–^127^ In particular, backmapping offers the possibility to include secondary structural changes of peptides and nucleotides, one of the major limitations of popular CG force fields like the Martini model. More challenging is the combination of AA and CG models in one system. Ideally in such an approach, the central part of the system is modelled at full detail, and the surroundings in CG resolution. Either a static or a dynamic approach could be followed. In the static approach, a predefined part of the system is modelled in all-atom, the rest in CG resolution. Pre-formed fibers and tubes could, in principle, be modelled in such a static hybrid fashion. Promising hybrid models coupling CHARMM and PRIMO,^128^ or any all-atom force field to Martini,^129^ have already been developed. In particular one would benefit from CGing the solvent as is done in the work of Riniker.^130^ In the dynamic approach, particles can change resolution on the fly as in the adaptive resolution scheme (AdResS).^131^ With respect to self-assembling systems, the AdResS approach is promising, but so far has been restricted to multiscale solvation of small (bio)molecules.^132^–^134^ One could imagine a system where the monomers in solution are represented at the CG level, and the growing aggregate(s) in full detail. However, such an approach faces two difficulties: first, the difficulty to extract the thermodynamic forces needed to compensate for the difference in chemical potential between the AA and CG models in all but trivial cases, and second, the non-optimal implementation of the AdResS method in available software packages, limiting the integration timestep to that of the all-atom model. Other challenges in the field of CGing are to build connections to continuum models (*e.g.*, of bundling fibres^135^) and to include chemical reactivity into the models, further expanding into the non-equilibrium field.

### Enhanced sampling techniques

Whether all-atom, coarse-grained or multiscale models are used, there is often a need for increasing the sampling of conformational space. Capturing supramolecular self-assembly in full requires not only sampling of the many ways individual monomers can pack together, but also sampling the overall morphologies available to hundreds or thousands of monomers as well as sampling of the manifold of kinetic pathways leading from one structure to the other. Several methods exist to increase the effective simulation time of MD simulations, by improving sampling of relevant areas of the assembly path. These methods operate by biasing the potential along a certain reaction coordinate or by effectively lowering energy barriers on the potential energy landscape. Popular methods that are applied in the field of supramolecular self-assembly include replica-exchange MD (REMD),^136^ meta-dynamics,^137^,^138^ steered MD^139^ and transition path sampling (TPS).^140^ Examples of these methods are provided below. For a recent review on enhanced sampling techniques, see ref. 141.

#### Structural convergence with replica-exchange

The most widely used enhanced sampling technique to study self-assembly is T-REMD. In T-REMD, temperature is used as reaction coordinate. In practice, multiple simulations (replicas) covering a predefined temperature range are performed in parallel. Configurations are swapped between the different temperature replicas, allowing energy barriers to be overcome. The power of T-REMD is illustrated by a study on Fmoc peptide fibrils with the OPLS force field.^142^ Many different ways of generating starting structures were compared. By using T-REMD, the authors could show a clear independence of the final structure, which in all cases converged to a condensed fibril structure in which the Fmoc groups stack mostly within the centre of the fibril. It was concluded that the stacking of the Fmoc groups, together with inter-residue hydrogen bonding and hydrogen bonding with water play important roles in stabilizing the fibril structure of supramolecular assemblies of short conjugated peptides. Other examples of T-REMD enhanced sampling include work on short peptides with an implicit solvent CHARMM force field^143^,^144^ and short peptides in explicit solvent modelled with OPLS.^145^,^146^ Replica exchange is not restricted to temperature as reaction coordinate. Another effective way to speed up the simulations is by tempering the solute interactions, *i.e.* by running replicas in which the solute–solute and/or solute–solvent interactions are weakened, so-called replica exchange with solute tempering (REST). Arefi and Yamamoto used this approach to study self-assembly of supramolecular BTA polymers in methylcyclohexane solvent.^147^ Their results show that both REST and T-REMD are able to predict highly ordered polymer structures with helical H-bonding patterns, in contrast to conventional MD which completely fails to obtain such a structure. In this case, REST proved more cost-efficient than REMD. Replica exchange can also be combined with other methods, such as umbrella sampling, to obtain free energies. Smith and Shell performed an extensive analysis of dimerization free energies of short peptides (Ser–Gly octamers, with systematic mutations of Leu), using an implicit solvent AMBER force field and umbrella sampling REMD.^148^

#### Finding new configurations with meta-dynamics

**Fig. 6:**
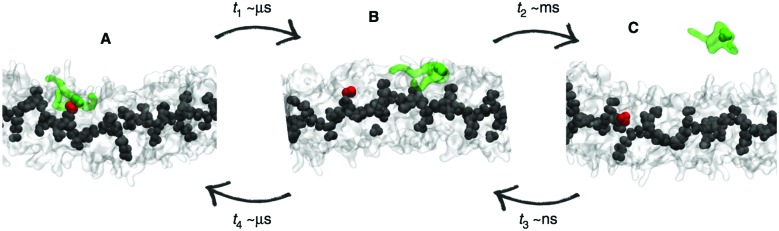
Enhanced sampling with meta-dynamics. Example of BTA fibre growth from Bochicchio *et al.*^151^ Starting from a configuration where the activated monomer (green in the snapshots) is stacked onto a hot spot (red) on the fibre surface (A), the monomer does not diffuse directly in water, but tends to diffuse onto the surface of fibre (B) before jumping in the solvent (C).

In meta-dynamics, energetic penalty terms are used in order to force the system to move away from the configurational space that has already been sampled. After the simulation, an unbiasing step allows to regenerate the unperturbed configurational space and provides access to the free energy. It is a powerful technique that can, for instance, be used to efficiently explore crystal packing and growth.^149^,^150^ In an exciting study of Bochicchio *et al.*, meta-dynamics was used to study the growth of supramolecular BTA fibres.^151^ The penalty terms forced the system to explore different scenarios for fibre growth, revealing a new growth mechanism in which monomer exchange in and out the fibres originates from the defects present in their supramolecular structure (Fig. 6). Analysis of the free energy changes involved further confirmed the feasibility of this pathway.

#### Mimicking experiments with steered MD

In steered MD, the system is driven in a particular direction using a biasing potential. Steered MD is often used to pull at a particular molecule in order to mimic AFM or optical tweezer experiments. The method has been used, for instance, to compute the interaction strength of peptides in monolayers^152^ or to study growth of PA fibres.^153^ More complicated reaction coordinates can also be used. Yu and Schatz used steered MD to force the transition between the bound and free states of 90 PAs (palmitoyl-Ser-Leu-Ser-Leu-Ala-Ala-Ala-Glu-Ile-Lys-Val-Ala-Val) in aqueous solution, with the bound state corresponding to a cylindrical micelle fiber.^154^ They considered the radius of gyration of the PAs as collective reaction coordinate to describe their collective assembly. They found that the PAs initially approach each other in mostly random configurations and loosely aggregate, resulting in significant desolvation and initiation of head–tail conformational reorganization. Subsequently, PAs undergo a conformational disorder-to-order transition, including forming secondary structures along with tail–head core–shell alignment and condensation that leads to total exclusion of water from the core.

#### Exploration of kinetic pathways with TPS

**Fig. 7:**
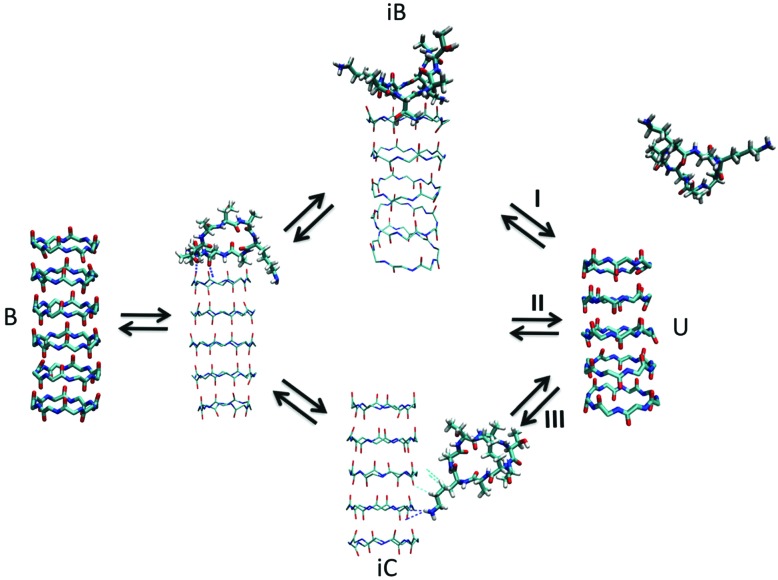
Kinetic pathways obtained with TPS. Example from Brotzakis *et al.*, showing three possible transition mechanisms found from bound (B) to unbound (U) states in the transition path ensemble of tube-forming cyclic peptides. Reproduced from ref. 155 with permission from The Royal Society of Chemistry.

To explore possible kinetic pathways, TPS is a suitable technique. TPS relies on the generation of new pathways connecting two states, by making random changes to an initial pathway. Using appropriate acceptance criteria, an ensemble of pathways is thus obtained with correct statistical weights between them. TPS has been used, for instance, to study dissociation pathways of tube-forming cyclic peptides with the all-atom AMBER force field.^155^ The study revealed multiple possible dissociation pathways (Fig. 7). TPS has further been applied to unravel the complex nucleation kinetics of short amyloidogenic peptides, modelled using an implicit solvent CG model.^156^

#### Challenges for enhanced sampling techniques

**Fig. 8:**
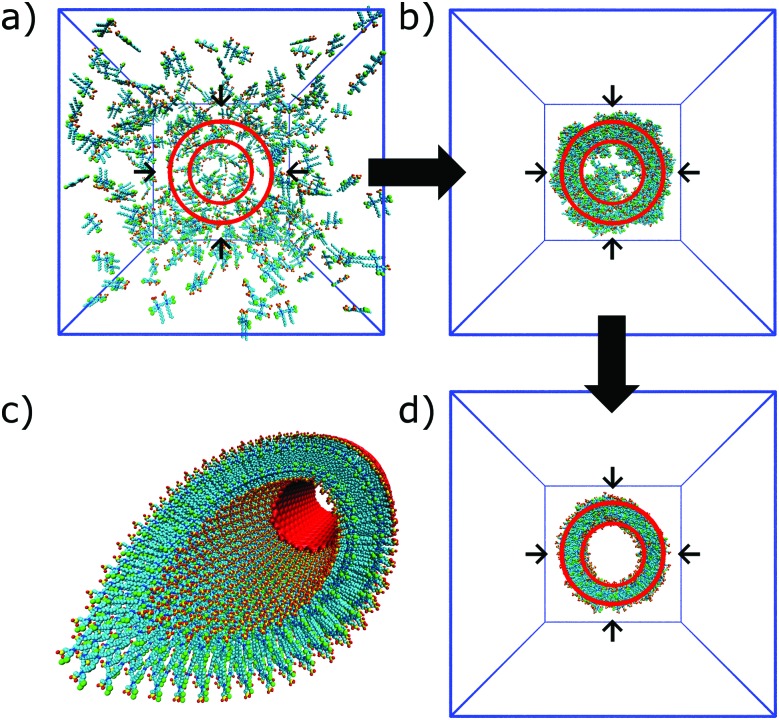
Self-assembly of C8S3 dye molecules into a double walled tube using cylindrical constraints. (a, b and d) Progression from randomly dissolved molecules towards the final tubular nanostructure using cylindrical constraints (indicated by the red circles). (c) Idealized C8S3 nanotube structure.

The potential variation in enhanced sampling techniques is almost unlimited. Main challenges for the near future lie in the combination of different methods. An example is the use of additional spatial constraints to bias the system in a smart way. This approach could be used in combination with meta-dynamics, as demonstrated by Limongelli and co-workers in case of reversible binding and unbinding of a ligand to a protein.^157^,^158^ Extension of this method to study adsorption and desorption of a monomer to a supramolecular polymer seems possible. Application of spatial constraints is also an effective means to bias the overall morphology of the system. For example, this approach has been used to speed up formation of lipid vesicles^159^ or to study the effect of a constraining volume on the type of aggregate formed by bolaamphiphiles.^160^ Effective constraints could, in principle, also be used to speed up self-assembly of bio-inspired molecules in tubes of fibers, as demonstrated by preliminary work from our lab on synthetic dyes (Fig. 8).^161^ Cylindrical restraints were used to drive assembly into a tubular aggregate that matches the size of the experimentally observed nanostructures. Other strategies to further improve sampling are the use of MC moves, as demonstrated in a number of studies on peptide aggregation.^162^–^164^

## Comparison of simulation and experimental results

Molecular simulations give a unique and often intuitive insight into the dynamics of self-assembled structures by explicitly showing the trajectory of every molecule in the system. Following this line of thought, many research papers have included simulations of the self-assembly process or the dynamics of an assembled structure. However, all too often their interpretation is left to the intuition of the reader and/or author, while knowing the position, velocity and acceleration of every particle in the system allows for quantitative comparisons with reality. The difficulty lies in the discrepancy between what can be measured in a laboratory (macroscale properties, ensemble averages) and what can be directly observed from molecular simulations, *i.e.* the motion of individual molecules. As the domains of simulations and experiments are converging more can be obtained from direct comparison (nanostructure dimensions and even self-assembly mechanisms), but indirect comparisons through quantum chemical calculations of spectra can be equally useful, especially when optical properties are available from the experiment. In this section, we review advances in both approaches.

### Direct comparisons of MD results with experiments

#### Distances and dimensions from microscopy and scattering data

Microscopy advances have rendered the shape and dimensions of supramolecular polymers the most obvious experimental observables. This can happen at intra-/intermolecular scales, or at the scale of the full nanostructure. In the first case, various groups compared H-bond or π–π stacked distances of the assembling biomolecules in an MD simulation to those from a wide-angle X-ray scattering or even single crystal X-ray structure^34^,^60^,^165^–^167^ or directly imaged 2D assemblies on surfaces using scanning tunnelling microscopy.^168^ However, such reliable data is not often available for soft self-assembled nanostructures as their dried crystal state is commonly different from their solvated state.^60^,^169^ In that case, the nanostructure dimensions can be measured and compared to microscopy or small-angle X-ray scattering (SAXS) results. For example, Yu *et al.* recently calculated the radial distribution function (RDF) of end-to-end distances of their PAs which matched the result from cryo-TEM and SAXS, when fitting to a core–shell cylinder model. Interestingly, SAXS and WAXS patterns can also directly be calculated from snapshots of MD simulations. This is already routinely done for large biomolecules and molecular liquids,^170^–^172^ but appears to be equally suitable for self-assembled nanostructures, especially (worm-like) micelles. As an example, Pedersen and co-workers recently successfully compared experimental and calculated SAXS patterns on dynamically formed complexes of lipid micelles and small proteins.^173^ Additionally, Pereira Oliviera *et al.* recently showed that MD simulations are able to elucidate the packing of cyclohexane tricarboxamide materials when combined with X-ray structural characterizations by studying the packing of individual 1D fibers.^174^ They managed to match computational and experimental intermolecular distances, but interestingly also demonstrated that the macrodipole created by stacking dipolar monomers in anisotropic materials can stabilize the materials themselves; a property often overlooked.

A property that shows up in many pre-assembled peptide or PA fibres is spontaneous twisting,^175^–^178^ also seen in experimental studies using AFM, TEM and CD spectroscopy. Lai, Rosi and Schatz simulated the formation of chiral nanofibers of a self-assembling gold-binding peptide conjugate, starting from achiral structures using atomistic MD and found the correct handedness and pitch.^175^ Frederix *et al.* took a similar approach and identified the handedness and pitch of stacks of peptide macrocycles.^54^ A still relatively unexplored area is the interaction between fibres. Bundling of fibres is observed in electron micrographs for many systems, but not easily studied due to the large system sizes involved. Initial attempts in this direction can be found in a number of publications.^41^,^179^,^180^ All these results combined suggest that interatomic distances, on both Å and tens of nanometres scale, can reliably be extracted from MD simulations.

#### Conformations from solid state NMR

**Fig. 9:**
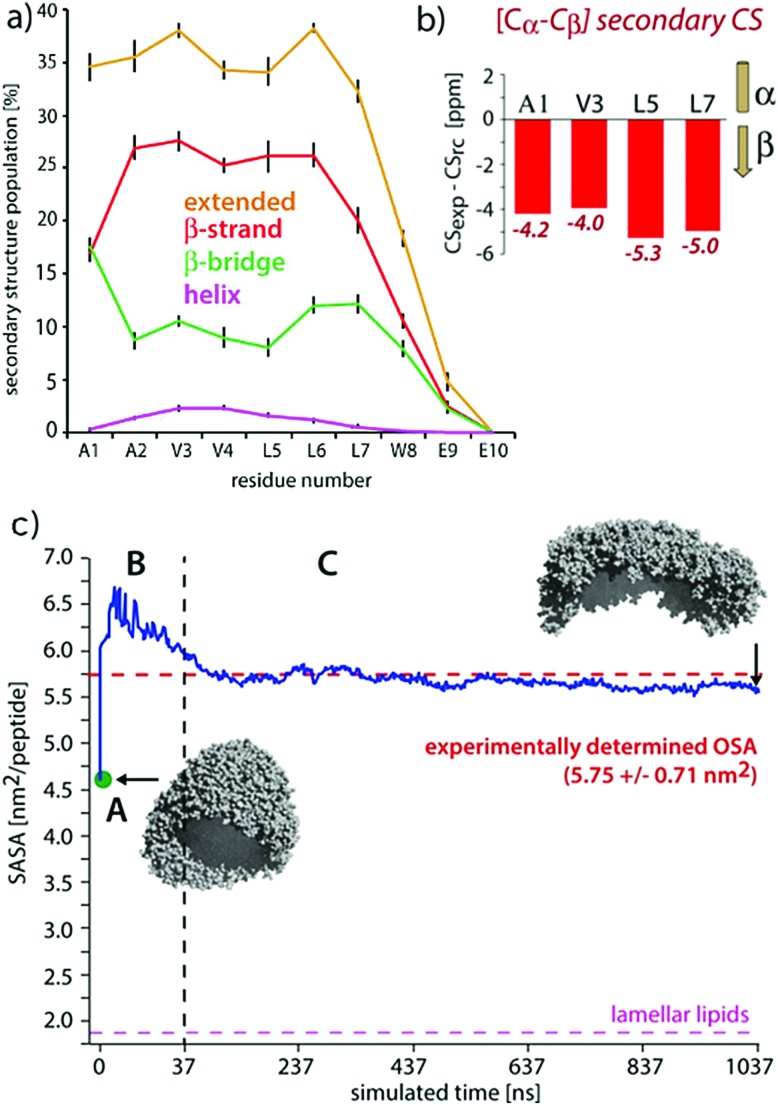
Comparisons of experimental and MD results on a peptide nanocarrier. (a) Residue-specific secondary structure extracted from a 1 μs atomistic simulation of 449 peptides. (b) Residue-specific ssNMR secondary chemical shifts indicating a full-β conformation. (c) Comparison of the occupied surface area (OSA) measured by static light scattering (SLS) and the solvent accessible surface area (SASA) derived from MD simulations. Point A (in green) is the starting system of the atomistic simulation (comprising 2500 SA2 peptides), back-transformed from the CGMD simulation. This system was evolved for 37.5 ns, shown in passage B. In passage C, an assembly of 449 peptides was further evolved for 1 μs. Figure adapted with permission from ref. 126. Copyright 2015 American Chemical Society.

Rad-Malekshahi and co-workers undertook a combined solid-state NMR (ssNMR) and static light scattering (SLS) approach to study their supramolecular peptide-based nano-carrier to high molecular details.^126^ Extensive atomistic and coarse-grain simulations were not only able to study the assembly mechanism and reproduce the decapeptide vesicle diameter, but also to measure peptide secondary structure and occupied surface area of the assemblies. The secondary chemical shifts from the ssNMR gave residue-specific secondary structure, while the surface area per peptide was determined by SLS. Both were found to be in excellent agreement with the simulations (see Fig. 9). Following a similar approach, Nagy-Smith *et al.* were able to determine the complete molecular structure of peptide fibrils in a hydrogel network.^181^ Perhaps surprisingly, ssNMR revealed that a β-hairpin peptide of interest to cell culture (MAX1) assembled in a completely monomorphic fashion. Various 2D NMR pulse sequences on isotopically labelled peptides informed distance and torsional angle restraints that were incorporated in atomistic MD/MC simulations. The combination of the two techniques made high level structural data available: MAX1 peptides assemble in a hairpin bilayer with a dry hydrophobic and interdigitated interface, but remain as individual fibers because the solvent face is strongly positively charged. Considering the usefulness of ssNMR to determine molecular conformations for biological peptide aggregates,^182^ we envision the popularity of this method to increase in bionanostructures in combination with MD simulations.

#### Dynamics probed using EPR spin labels

**Fig. 10:**
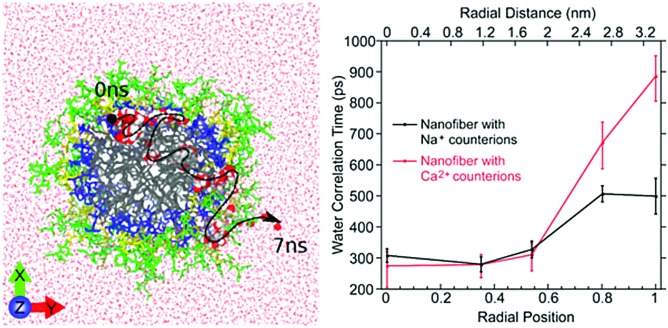
Left: Atomistic simulation snapshot illustrating the trajectory (dashed line) of one water (highlighted) diffusing across the nanofiber in around 7 ns simulation time. Right: Experimentally measured water correlation time as function of the distance from the nanofiber core. Figure reproduced with permission from ref. 45. Copyright 2017 American Chemical Society.

The groups of Stupp and Han recently developed experiments to probe the internal freedom and dynamics of PAs nanostructures using site-directed spin labelling and electron paramagnetic resonance (EPR) spectroscopy^183^ and Overhauser dynamic nuclear polarisation (ODNP) relaxometry on water.^45^ Both techniques rely on chemical modification of the self-assembling building block to include a nitroxide radical spin label. In the case of EPR spectroscopy, the rotational diffusion of the labelled site is a direct probe for the internal dynamics of the amphiphiles. It was thus shown that amino acid residues in β-sheet conformations display significantly slower, solid-like rotational diffusion, while other amino acids and the aliphatic core of the supramolecular assembly display a viscous liquid behaviour (Fig. 10). As a saturated EPR spin can undergo cross-relaxation to ^1^H nuclear spin, the relaxation of the hyperpolarisation on water hydrogens can be measured, effectively extracting water dynamics only in the proximity of the spin label. The water correlation times thus measured and calculated from atomistic MD simulations both showed much faster water dynamics in the hydrophobic core of the PA fibres than in its peptide region, where its motion resembled that of water in protein cavities.

### Indirect quantitative comparisons

#### Peptide secondary structure through infrared absorption

The spatial resolution of most experimental techniques is not high enough to resolve individual (parts of) molecules when they are part of a nanostructure. Intermolecular interactions and molecular conformations are therefore probed using spectroscopic or scattering techniques. For example, Fourier-transform infrared (FTIR) spectroscopy is routinely used to determine the secondary structure of proteins, as the absorption frequency of the backbone amide groups in the amide region of the spectrum (1600–1700 cm^–1^) is strongly dependent on H-bonding and the neighbouring amide moieties *via* transition dipole coupling (TDC). This physical effect leads to signature bands for specific secondary structure elements.^184^–^186^ When bioinspired supramolecular materials contain a peptide component FTIR or multidimensional IR techniques can give similar insights into their conformation, although QM calculations and TDC models have shown that the interpretation for proteins does not necessarily hold for short peptide chains.^187^,^188^

**Fig. 11:**
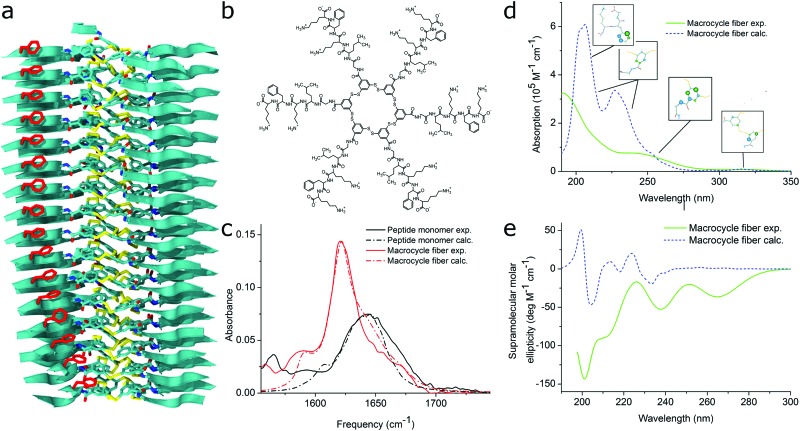
MD simulations and spectral calculations on self-assembled self-replicating peptide macrocycles. (a) Chiral fiber nanostructure from MD simulations. Peptide chains are indicated as ribbons. (b) Macrocycle chemical structure. (c) Experimental and calculated linear infrared spectra of the fiber. (d) Experimental and calculated UV/Vis spectra of the fiber. The location of the transition dipoles for each of the calculated bands are indicated by the insets. (e) Experimental and calculated supramolecular CD spectra of the fiber. Figure adapted from ref. 51.

Density functional theory has been successfully used to determine the vibrational structure of small amphiphiles clusters,^110^,^187^ but does not capture long-range coupling and the effect of disorder very well due to its static structure. While DFT-MD has been applied to single or dimers of peptides to resolve the flexibility issue,^189^ calculating the full electronic structure of a nanostructure of 100s of monomers evolving over time is computationally unattainable. The work of many groups, recently reviewed by Reppert and Tokmakoff.^190^ has overcome this issue using a mixed quantum-classical approach that uses frequency mapping^191^–^195^ and TDC theory^196^–^198^ while representing amide groups as environment-dependent coupled oscillators. Experimentally, the groups of, among others, Zanni and Woutersen have studied short various biological fragments of amyloidogenic proteins (*e.g.*ref. 199 and 200) and shown the power of combining (ultrafast) infrared spectroscopy and calculations for short peptides. The first use specifically for self-assembling designer PA was recently reported by Frederix *et al.* who studied the self-assembly of self-replicating peptide macrocycles using both experimental techniques and semi-empirical calculations on MD trajectories (see Fig. 11).^54^ They found excellent agreement between the calculated spectra of a dynamic 1D β-sheet-like fiber and the experimental result. By averaging over many MD frames and saving snapshots with approximately the same frequency as the vibrational period, both the homogeneous and inhomogeneous line broadening of the IR transitions, respectively, are part of the calculation output. This advantage over QM calculations is particularly useful in time-resolved 2D spectra, but also in linear spectra to indicate whether the degree of disorder in the structure is accurately represented by the simulated structure.

Other regions in the IR spectrum can also help in interpreting the dynamics of nanostructure as shown by Deshmukh *et al.* for the water OH stretch region.^125^ Vibrational spectra of both bulk water and water molecules close to the PA nanofibers were calculated by Fourier transforming of the atomic velocity autocorrelation function obtained from the MD simulation trajectories. This showed much more ordered water molecules in proximity to (especially hydrophilic) PA groups, which was experimentally validated by comparing PA assembly kinetics by IR and CD experiments in H_2_O and D_2_O.

#### Electronic structure through UV/Vis and CD spectroscopy

UV/Vis, fluorescence and CD spectroscopy are routinely invoked to probe any change of environment of conjugated moieties of the self-assembling building block. Assembly of π-systems generally lead to shifts in absorption spectra and can completely quench or enhance fluorescent emission. Note that we are mainly interested in the inherent absorption or fluorescence of the self-assembling moiety. The introduction of dyes such as Nile Red or Thioflavin T is also commonly used to label hydrophobic or β-sheet regions, respectively, but these are typically at low concentrations and considered orthogonal to the assembly process and therefore not included in the simulations.

Analysis of experimental electronic spectra often occurs on a qualitative level without clear information on the underlying physical principles. This originates from a historic lack of computational power to unravel the electronic structure of large non-covalent aggregates containing a lot of disorder, while the theory on J- and H-aggregates is reasonably well-developed for small complexes. The multitude of vibronic states in supramolecular polymers also complicates matters further because multiple conformations can produce similar spectra when broadened to a large extent by disorder. In order to obtain a quantitative description and information on the molecular arrangement of monomers inside the aggregates, theoretical methods have been employed to model their optical electronic states.^201^–^203^ More specifically, exciton Hamiltonians have been used to describe the excitonic states of the system. The basic forms of the Hamiltonians usually include two parameters, one for the molecular electronic excitation and another one for the excitonic coupling of different monomers. Diagonalization of the Hamiltonian operators produces the simulated optical spectra of the aggregates. The electronic coupling and especially the CD signal is often very sensitive to disorder in the molecular packing as theoretically shown by Van Dijk *et al.* and quantitatively analysed and reviewed by Pescitelli, Di Bari and Berova.^204^–^206^ This emphasizes the importance of using MD simulated input structures rather than idealized putative structures. In many cases, the simulated optical spectra present good agreement with the experimental measurements when disorder is taken into account.^43^,^207^ Also note that the CD signal calculated by exciton methods typically covers only the supramolecular component of the signal and molecular chirality should be subtracted. However, many studies have demonstrated that the induced CD signal by assembly is generally significantly different or larger than the molecular component, such as for protein secondary structure determination^208^ and achiral molecules forming helical assemblies.^209^–^212^ Alternatively, the molecular CD signal can be extracted from experiments below the critical aggregation concentration or above the critical aggregation temperature.

For self-assembling bio-inspired molecules, electronic structure calculations were recently used to understand the UV/Vis and CD spectra of the self-replicating peptide macrocycles described above (see Fig. 11).^54^ UV/Vis and CD spectra calculated from MD structures displayed reasonable agreement with experimentally measured spectra, validating the proposed structural models for supramolecular packing. Importantly, the chirality of the nanostructure was appropriately represented by the simulations: both the helical pitch and the handedness (and thus sign of the supramolecular CD signal) were accurately predicted by the combined MD simulations and electronic calculations. Additionally, assignment of the absorption bands to specific chemical moieties in the building block provided crucial information for the assembly mechanism by exploring the behaviour of the bands during T-induced disassembly of the nanofibers.

**Fig. 12:**
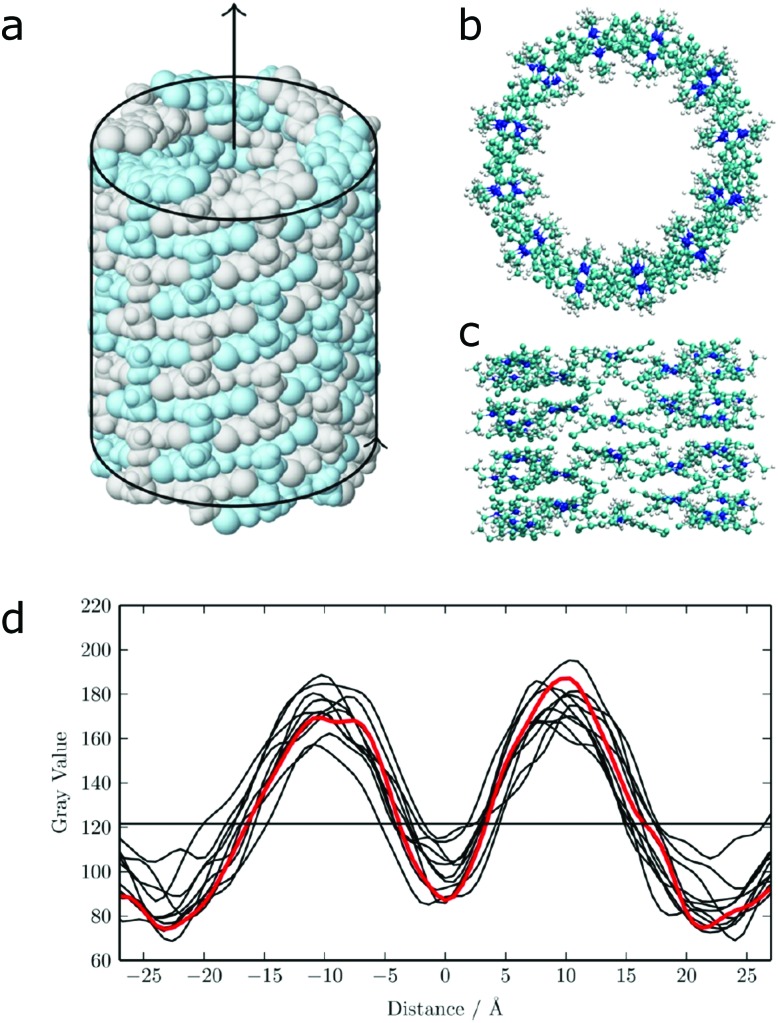
(a) Idealized model for a nanotube structure of TTBC. (b) Top and (c) side view of the tube structure after MD simulations. (d) Comparison of the experimental density profile from cryo-TEM (red) with simulated 2D density profiles from 10 different angles (black lines) of the MD structure. Figure adapted from ref. 43.

The calculation of optical properties is of relevance for the structural analysis of self-assembled structures as outlined above, but can also be of inherent significance to the function of the structure. Such is the case in supramolecular light-harvesting structures, such as dye aggregates that mimic natural photosynthetic systems or can be used in organic photovoltaics. A well-studied class of such aggregates is cyanine dyes, which optical properties are determined by the polymethine chromophore (Fig. 1 and 12). Their behaviour in solution and aggregates can be tuned using different substituents such that well-ordered nanostructures are formed as evidenced by cryo-TEM.^43^,^201^,^213^,^214^ In principle the spontaneous assembly of such systems can be studied using MD simulation, as evidenced by Haverkort *et al.*^41^ An amphiphile pseudoisocyanine dye, amphi-PIC, was studied by MD simulations and two types of aggregates, single-walled tubes and ribbons were generated. However, it was found that the final arrangement of the self-assembled structure lacks long-range order. Megow and co-workers therefore studied similar (C8S3) cyanine dyes starting from preformed nanotubes instead of a spontaneously assembled structure. The nanotubes remained stable for approximately 7 ns, although they disintegrated in time afterwards. The simulated absorption spectra for the obtained MD structures resulted in good agreement with the experimentally measured spectra, when the dispersive energy shifts of the molecules in the outer and the inner wall were taken into account in the theoretical models for the optical properties of the nanotubes.^44^ Similarly satisfying results were obtained from MD structures of other dye aggregates, where theoretical models, experimental measurements and computational studies have been combined together.^43^,^215^ These results indicate that the developed systematic procedure for structure prediction of self-organized tubes cyanine dyes is generally valid.

## Conclusions and outlook

This review discusses the important developments in obtaining insight into the self-assembly of bio-inspired molecular systems using classical MD. On the one hand, advances in force fields and enhanced sampling methods improve the speed and accuracy with which useful information can be obtained from simulations. On the other hand, new experimental techniques and methods to directly relate their results to computational output enhance the synergy between the two traditional realms. To advance our understanding further, we identify several challenges for the field that we expect to accelerate major improvements in the future: the convergence of simulated and experimentally accessible time and length scales, far-from-equilibrium systems, computational model improvement and the rational design of *de novo* supramolecular systems.

### Convergence of time and length scales

Self-assembly takes place on both the smallest and largest time and length scales. Simulations are currently the main method to study (the aggregation of) individual molecules, but experimental developments are also going more and more towards single-molecule resolution. Single-molecule spectroscopy has seen great improvements in the last decade, especially in determining the position, structure and dynamics of fluorescent or fluorescently labelled molecules. Microscopy techniques have advanced simultaneously to have better time resolution (*e.g.* high-speed AFM has sub-second time resolution at a few nm spatial resolution and has in some cases revealed the mechanism of supramolecular polymerization^112^,^216^) or better spatial resolution (*e.g.* super-resolution microscopy has been used to follow both monomer mixing and self-sorting in supramolecular polymers^48^,^217^). Another promising technical advance is the development of (nano- and) microfluidics, which has already enabled the detailed study of the anisotropic mechanism of the supramolecular polymerisation of diphenylalanine.^218^,^219^ Spatial confinement and control of the chemical potential of building blocks in solution crucially allows for the detection of intermediates in the self-assembly process which are difficult to capture otherwise.

With experiments going towards smaller and shorter scales, molecular simulations are going the other direction to meet them in the middle. Both hardware and software developments, together with more accurate coarse-graining techniques are allowing longer simulations of larger systems. Automated parameterisation algorithms, when used with care, can strongly reduce the effort to study new self-assembling systems. Enhanced sampling techniques allow a faster and more complete search of the conformational space available. Naturally, when computational and experimental techniques are able to probe the same molecular processes directly, the confidence in any conclusions deriving from the work is greatly enhanced. For example, some ensemble spectroscopic techniques are being revisited for use in supramolecular chemistry. NMR was long deemed unsuitable for large aggregated structures due to their lack of tumbling in solution, but recently developed protocols for ssNMR have determined the molecular architecture of supramolecular assemblies to almost single-crystal resolution.^181^,^182^,^220^ Technical and theoretical developments in vibrational CD experiments are starting to allow the absolute chirality and organisation of various biomolecular supramolecular architectures to be studied.^221^ We have not explored calorimetry measurements in this review, but work from the Bouteiller group and others has shown that the thermodynamics of monomer association to a supramolecular polymer can be revealed by isothermal titration or differential scanning calorimetry.^222^ For the case of BTA, this was combined with MM/MD simulations to get a complete picture of the self-assembly energetics,^223^ but would ideally be compared to free energy calculations from, for example, umbrella sampling, meta-dynamics or TPS simulations that are more accurate in determining the potential energy landscape.

### Steering the pathway: away from equilibrium

The energy landscape of the self-assembly process is, in fact, one of the major subjects of investigation in the supramolecular chemistry field. In an effort to make dynamic, functional and life-like materials, that can self-heal or are responsive, systems need to be able to continuously convert an energy input (*e.g.* chemical, mechanical) to remain away from thermodynamic equilibrium.^224^,^225^ This presents a serious challenge for both experimental and computational studies of self-assembling systems: while it has been shown that supramolecular fibers can represent the thermodynamic minimum,^226^ in most cases assembled structures represent kinetically trapped states, and the outcome of a self-assembly experiment or simulation therefore depends on the pathway followed.^227^–^230^ This implies that a large portion of the multimeric conformational space needs to be sampled to address all possible routes towards a final nanostructure. Techniques like TPS have started to open up this possibility, but require knowledge regarding the start and ending structures, which may not always be available. Recently, experiments and kinetic models have been developed to describe the thermodynamics for short peptide assembly,^231^ which crucially provides information on kinetic barriers and state equilibria. MD simulations and free energy calculations have great potential to work together with these kinetic equation-based approaches and link these barriers, for example, to actual supramolecular processes. The ultimate challenge would be to control the self-assembly pathways not only globally, but also locally, *e.g.* using compartmentalisation techniques, laser induced local heating, or stressing and steering with molecular motors. From a theoretical point of view, the aim is to mould the underlying energy landscape in a controlled way in order to bias the propensity of a system to end up in certain states, or to allow or disable certain response directions by adding or removing kinetic barriers. In the end, a non-equilibrium thermodynamic framework is needed to guide simulation efforts in this direction.

### Computational model improvement

We expect that improvements in the (semi-empirical) quantum mechanical description of supramolecular systems will drive the comparison of simulated and (usually easily accessible) experimental results further. The prediction of IR spectra discussed in the previous section has seen many successes specifically for the amide I band of the spectrum, but an abundancy of self-assembling systems contain other functional groups that are potential markers for structure. Except from brute-force DFT calculations, theory is still lacking to describe vibrational coupling in this case. Electronic calculations are usually more flexible considering functional groups, but the computationally relatively cheap methods that allow using the large number of molecules in an assembly, such as ZINDO/S,^232^ were developed for and fitted on small organic molecules with mainly single-electron excited states and a small active space, and perhaps should be recalibrated for systems of many molecules with near-degenerate excited states.

Finally, we want to note that some of the major successes in describing self-assembly using MD have taken the supramolecular behaviour of the system into account already in the parametrization stage. Indeed, small deviations from reality on the monomer level can be strongly amplified by having hundreds of interacting monomers. For example, some MD force fields suffer from overestimation of protein–protein interactions, because the individual components (amino acids) have been parametrized towards protein stability. This can be solved by including the nature of the assembly, *e.g.* nucleation–elongation kinetics as a target property. Solutions may include polarisable force fields as discussed above, but can be included as a target in classical MD as well, as shown by Bochicchio and Pavan and our lab.^111^,^112^

### Toward rational design

In the previous sections we have discussed successes in validating nanostructures proposed based on experiments or even putative structure relying on chemical intuition. However, as the chemical space for bio-inspired nanostructures is enormous, it would be particularly interesting to simplify the effort of discovering new bionanostructures by predictive computational approaches. The rational design of small to medium-sized building blocks that assemble has seen many successes from the field of foldamers,^212^,^233^–^236^ single molecules that fold *via* non-covalent interactions, often through intramolecular recognition motifs using similar interactions as in the general field of self-assembly (H-bonds, π-stacking *etc.*). The challenge ahead now is the prediction of the packing of many molecules together in larger nanostructures (dimensions greater than a single foldamer). MD assembly simulations can be used to screen among self-assembly candidates in a high-throughput fashion and one can imagine this approach can be extended to other factors commonly affecting self-assembly, such as pH, and ion nature and concentration.

The above considerations point towards a change in mind-set in supramolecular chemistry. It is even argued to abandon the term “self-assembly”, which implies a passive approach, in favour of “non-covalent synthesis”, where the scientist does not only choose a building block to start with, but designs a complete bottom-up route to get from individual components to a functional nanostructure. We conclude that directly applicable input from simulations will prove decisive in speeding up this transformation.

## Conflicts of interest

There are no conflicts to declare.
